# A Study on Condition-Based Maintenance for Wafer Table Edge Degradation in Photolithography Equipment

**DOI:** 10.3390/s26123650

**Published:** 2026-06-08

**Authors:** Kyunghwan Joo, Kwang Hoon Lee, Jae Wook Jeon

**Affiliations:** 1 Department of Semiconductor and Display Engineering, Sungkyunkwan University, Suwon 16419, Republic of Korea; 2 Samsung Institute of Technology, Yong-in 17113, Republic of Korea; 3 School of Chemical and Biological Engineering, Seoul National University, Seoul 08826, Republic of Korea

**Keywords:** condition-based maintenance, process control, defocus monitoring, focus metrology, degradation

## Abstract

**Highlights:**

**What are the main findings?**

- Edge-Resolved Metrology: A novel monitoring methodology was proposed by utilizing existing Geometry-based Optical Focus Metrology (GOFM) technology. This approach determines the degradation level of the wafer table by isolating the pure focus residual specifically within the extreme outer edge (140–147 mm radius) of 300 mm wafers during photolithography operations.
- Dual-Indicator Optimization: A mathematical dual-indicator framework was optimized by integrating the Range Percentile 97% (statistical) and Slope × 3 (geometric). This framework successfully screens out baseline measurement noise to capture actual physical wear.

**What are the implications of the main findings?**

- Condition-Based Maintenance System: An automated Preventive Maintenance (PM) architecture was constructed based on a simple OR-logic protocol. The system directs immediate maintenance interventions if either indicator reaches its limit.
- Yield and Uptime Maximization: Shifting from routine time-based maintenance to a preemptive trigger at the quality-based critical warning threshold fundamentally prevents severe Critical Dimension (CD) anomalies. This direct intervention increases overall tool availability and extreme edge yield.

**Abstract:**

This study proposes a condition-based maintenance monitoring method based on Geometry-based Optical Focus Metrology (GOFM) to detect wafer table edge deterioration early and enable proactive interventions before actual Critical Dimension (CD) bridge defects occur. In advanced Deep Ultraviolet (DUV) immersion photolithography, prolonged equipment operation mechanically wears the wafer table, inducing Edge-Roll-Off (ERO). Because conventional optical metrology struggles to separate this localized defocus from process noise, this work utilizes the existing GOFM technique to isolate the pure focus residual within the 140–147 mm radius region. To quantify this hardware-specific degradation, a mathematical dual-indicator system was constructed. This framework integrates a statistical threshold, the Range Percentile 97%, to reject baseline measurement noise, and a geometric variable, Slope × 3, to capture the topographical drop in the outermost 3 mm. Analysis of long-term time-series data from multiple High-Volume Manufacturing (HVM) scanners confirmed a strong correlation ($R^{2}=0.93$) between these indicators. Furthermore, we proved that the drift trajectory of Slope × 3 deterministically predicts mechanical failure prior to defect occurrence on production wafers. Based on these findings, an automated condition-based maintenance architecture was designed using an OR-logic decision gate. By triggering a preemptive table replacement at a quality-based critical warning threshold, this system converts routine time-based scheduling into a data-driven paradigm, maximizing both edge yield and equipment uptime. Furthermore, this proposed framework establishes a solid foundation for future extensions toward machine learning-based predictive maintenance.

## 1. Introduction

As the continuous miniaturization of devices progresses in the semiconductor manufacturing process, various technologies such as Optical Proximity Correction (OPC) and Resolution Enhancement Techniques (RETs) have been widely introduced to overcome the limit of resolution [1,2,3]. Through this, DUV immersion lithography technology has continuously widened the limit of resolution, but as the Numerical Aperture (NA) reached its physical limit, the available Depth of Focus (DoF) rapidly decreased [4,5]. As a result, maintaining focus stability and precisely measuring it to secure a uniform CD across the entire area of a 300 mm wafer, especially in the edge region where geometric distortion and mechanical stress are concentrated, has become the most core task in modern semiconductor manufacturing [6,7,8,9]. The latest high-productivity exposure equipment adopts a dual-wafer table system.

**Figure 1:**
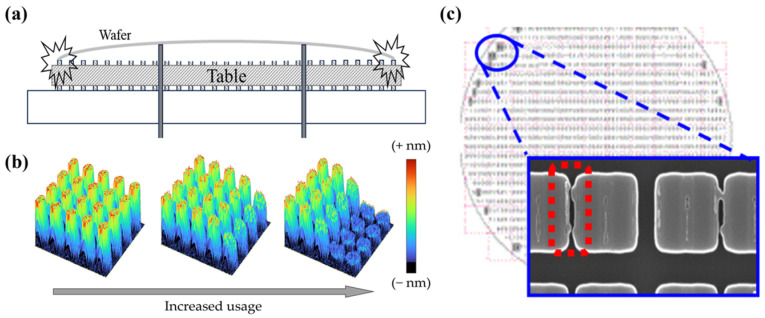
Mechanism of wafer table edge wear and its impact on photolithography patterning quality. (**a**) Mechanism of water table and warpage of wafer. (**b**) Extreme outer burl wear progression according to the increased usage. (**c**) Representative top-down SEM image of a fatal pattern bridge due to table wear.

However, as the wafer loading and unloading cycles are repeated for a long period, physical friction is continuously applied to the burl structure at the extreme outer edge of the wafer table due to the difference in clamping pressure and vacuum degree between the center and the edge of the wafer [10]. This mechanical wear causes a rapid drop in the wafer topography, that is, the ERO phenomenon. ERO induces a localized and severe negative defocus at the edge of the wafer, and this becomes the direct cause of pattern bridge defects in the edge region, linewidth nonuniformity, and ultimately edge yield degradation (Figure 1) [11,12,13,14,15].

Despite the severity of the problem, existing focus management technologies have clear limitations in detecting and monitoring the mechanical wear of the wafer table in advance. Previously, offline measurements using CD-SEM or leveling sensors built into the equipment have been mainly utilized [16]. Recently, Diffraction-Based Focus (DBF) technology was introduced to HVM, but preceding studies have mostly focused only on maintaining the best focus of the wafer center or shot unit [17,18,19,20,21]. In other words, studies that separate and quantify the localized ERO defocus, which rapidly occurs within 1–3 mm of the extreme outer edge of the wafer due to the mechanical aging of the equipment, in an edge-resolved manner are still insufficient.

Maintenance practice in advanced manufacturing has been progressively shifting from time-based scheduled servicing toward Condition-Based Maintenance (CBM) and Predictive Maintenance (PdM), in which equipment health is inferred directly from real-time sensor and metrology signals [22,23,24,25]. Within semiconductor manufacturing, this shift has been accelerated by machine learning (ML)-based fault diagnosis and Remaining Useful Life (RUL) estimation, ranging from stacked classifiers and autoencoder-based anomaly detection to recurrent and Transformer-based time-series models, now actively explored across diverse process tools [26,27,28,29,30,31,32,33]. In parallel, edge-resolved focus and overlay metrology have advanced through diffraction-based and scatterometry-based techniques that enhance measurement density and signal sensitivity at the wafer periphery, as highlighted in the 2024 IRDS Metrology roadmap [34].

The convergence of these two streams, however, has not yet reached the specific failure mode addressed in this work, namely mechanical wear at the extreme outer edge of the wafer table in 300 mm DUV immersion scanners. A data-driven PdM model for this failure mode requires both a physically interpretable degradation indicator validated as a leading marker of CD-level yield loss and a sufficiently long-term, consistent metrology dataset to support supervised learning, neither of which has been systematically established in the prior literature for wafer table edge wear [35,36]. This study therefore deliberately positions itself at the CBM stage of the CBM-to-PdM maturation pathway, constructing a deterministic, physics-grounded dual-indicator framework that links hardware-resolved focus metrology to maintenance decision-making and thereby establishes the empirical foundation on which subsequent ML-based RUL estimation can be built, as further outlined in Section 4.4.

To overcome this gap in the existing studies, this study introduces the GOFM technology utilizing the Optical Diffraction Metrology Mark (ODMM) designed on the reticle. GOFM is an innovative metrology method that converts it into a high-sensitivity focus error signal by measuring the ±1st-order diffracted light intensity difference reflected from the asymmetric Side Wall Angle (SWA) of the exposed pattern with scatterometry [37,38]. Through this, in-line focus data can be collected with an overwhelming density compared to existing methods.

The core contribution of this study lies in extracting the wafer table focus residual, from which equipment and reticle offsets are mathematically removed based on GOFM measurement data, and newly proposing two independent yet complementary wear indicators (dual-indicator framework) that monitor edge wear based on this. First, we derived the Range Percentile 97% indicator, which statistically represents the macroscopic focus degradation distribution of the entire 140–147 mm edge region even amid extreme measurement noise. Second, we defined the Slope × 3 indicator that quantifies the geometric bending slope of the ERO topography in the extreme outer 3 mm section of 144–147 mm where physical friction is most concentrated.

This paper verified its robustness and reproducibility by applying the proposed dual-indicator monitoring system to multiple exposure equipment in an actual HVM environment through year-based long-term monitoring. This study possesses profound industrial and academic significance in that it goes beyond investigating edge focus degradation and pattern defect mechanisms and lays the foundation for Condition-Based Maintenance (CBM) that can preemptively intervene before wafer table wear reaches a fatal critical threshold. Furthermore, this framework serves as a fundamental stepping stone toward advanced predictive maintenance by establishing data-driven indicators that can incorporate future machine learning models for forecasting RUL.

## 2. Materials and Methods

### 2.1. Lithography Tool and Process Conditions

All experiments and data collection of this study were performed using 300 mm DUV immersion photolithography equipment operating in an actual advanced memory semiconductor HVM line. The corresponding equipment is equipped with a dual-wafer table system that simultaneously performs measurement and exposure. To precisely track the physical wear mechanism of the mass production environment, year-based long-term monitoring was conducted targeting an HVM environment processing thousands of wafers per day.

The optical and process conditions used in the experiment applied the same Process of Record (POR) standard as the critical layer patterning of highly integrated memory devices. For the main illumination system conditions, annular and quadrupole modes were used in combination, and the NA was set to a maximum of 1.35, which is the default hardware specification and industry-standard configuration for the targeted ArF immersion scanner. For focus measurement wafers, a flat bare Si-type Non-Production Wafer (NPW) was used to exclude the noise of the surface topography. The detailed experimental parameters to guarantee the reproducibility of the study are summarized in Table 1.

### 2.2. GOFM and ODMM-Based Focus Metrology

**Figure 2:**
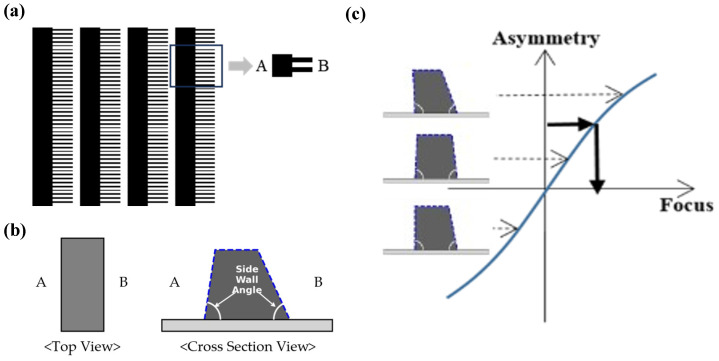
Basic principle and operation flow of GOFM. (**a**) A planar schematic diagram of the ODMM designed on the reticle. (**b**) A cross-section SEM image of the pattern formed on the wafer after exposure. (**c**) Correlation curve between focus variation and structural asymmetry.

To detect minute surface topography changes due to the mechanical wear of the wafer table, the GOFM technology was utilized. The core of the GOFM measurement is the ODMM etched on the reticle. As shown in Figure 2a, the ODMM was designed to have a normal bar pattern on one side and a sawtooth-shaped sub-resolution structure smaller than the resolution limit of the exposure equipment on the opposite side.

When this mark is exposed on the wafer, the SWA on the left and right of the pattern collapses asymmetrically due to the optical diffraction characteristics (Figure 2b). As the focus deviates from the best focus during exposure, the asymmetry of this SWA monotonically increases. Thereafter, the scatterometry equipment irradiates light onto this pattern, measures the difference in the ±1st-order diffracted light intensity, and converts this into a focus error value having a precision of a few nanometers through a mathematical algorithm (Figure 2c) [39].

**Figure 3:**
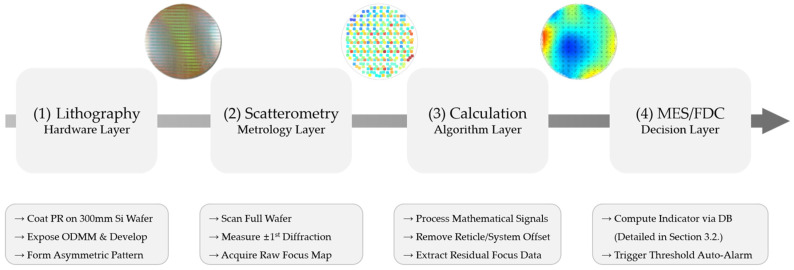
Integrated data pipeline of the GOFM-based mass production wafer edge monitoring system.

In this study, after exposing thousands of ODMM patterns on the entire surface of the NPW with high density, an integrated metrology framework that extracts focus data through In-line scatterometry and accumulates this in real-time to the central Manufacturing Execution System (MES) server was constructed (Figure 3). This framework is composed of 4 continuous steps from the equipment layer to the decision-making layer. First, lithography: through PR coating, ODMM reticle exposure, and development on the NPW [40], a diffraction asymmetric pattern is formed. Second, scatterometry: by optically scanning the entire area of the wafer, the ±1st-order diffracted light intensity is measured, and this is converted into a high-density Raw Focus Map. Third, calculation: by mathematically calculating the acquired signal, the reticle and system offsets are subtracted, and the Residual Focus data unique to the pure wafer table is extracted. Fourth, MES/FDC: Through the central server database, indicators (refer to Section 3.2 for details) are calculated, and upon reaching the threshold, an alarm for condition-based maintenance is automatically triggered [41].

### 2.3. GOFM Performance Metrics and KPI Definitions

The measurement performance of the GOFM-based focus metrology was defined and evaluated centering on the following four core KPIs.

(1) Focus Capture Range: It was defined as the focus range where the focus and asymmetry maintain a monotonic relationship and are matched 1:1. Experimentally, by optimizing the ODMM pitch condition, it was designed so that the capture range exceeds the minimum operational requirement for high-volume manufacturing, and if it deviates from this range, it was considered an area where focus measurement is impossible.
(2) Set/Get Residual: It was defined as the difference between the input focus offset and the focus value measured by GOFM. After exposing the wafer under various focus split conditions, by measuring the residual distribution of the focus value measured for each condition and the input value, the system was aligned and calibrated so that the set/get residual met the stringent tolerance threshold required for critical layer patterning.
(3) Zero Asymmetry Focus (ZAF): It was defined as the distance from the nominal focus to the focus where asymmetry becomes 0. Since a smaller ZAF value means that the GOFM signal operates more robustly against focus error-inducing factors such as lens aberration, resist height, and process stack change, it was designed and tuned targeting a minimized value below the acceptable process baseline.
(4) Sensitivity/Linearity (R^2^): It was defined as the sensitivity and linearity indicator (R^2^) representing how accurately and linearly the measurement signal can track the actual focus variation amount. As the value of this indicator is closer to 1, it means that the measurement reliability and the sensitivity to focus error are excellent, and it was evaluated with the goal of securing an overwhelming linearity compared to the existing method.

The above KPIs were used as benchmarking indicators with the existing eDoF (exact Depth of Focus)-based focus monitoring, while simultaneously quantifying the measurement performance of GOFM itself. The eDoF-based approach is a conventional focus monitoring method in which exposed wafers are evaluated by an external optical metrology tool, rather than by any scanner-integrated sensor, to determine the actual usable depth-of-focus window of the process. However, its limited number of measurement points and substantial point-to-point dispersion preclude reliable resolution of the extreme outer edge region (R ≥ 140 mm) where mechanical wear-induced ERO is concentrated, which motivates the GOFM-based high-density edge-resolved comparison performed in this study. In particular, by comparing the capture range, set/get residual, ZAF, and sensitivity of the eDoF-based focus method and GOFM under the same equipment and process conditions, the relative advantages in terms of focus error margin in the edge region, measurement stability, exposure/measurement time reduction, and monitoring automation were evaluated.

### 2.4. Definition of Wafer Table Focus Residual

Raw focus data captured by GOFM contains the wafer table topography alongside systemic offsets. These artifacts include reticle manufacturing flaws, global scanner tilt, and field-level lens aberrations. A mathematical correction protocol separates these system components to evaluate the table-driven ERO at the extreme periphery.

**Figure 4:**
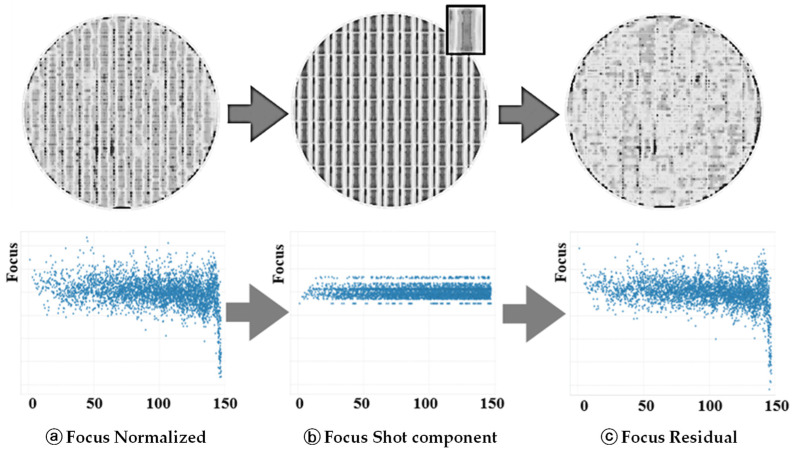
Mathematical derivation of the wafer table focus residual from raw GOFM data. ⓐ Focus normalized = focus raw—focus mean (of all measured points). ⓑ Focus shot component = focus fitting by 2D interpolation (by each field). ⓒ Focus residual = ⓐ focus normalized − ⓑ focus shot component → use only edge points of ⓒ focus residual for edge-wearing control.

In this study, as presented in Figure 4, the focus shot component was calculated by fitting the system error originating from the equipment and reticle with 2D interpolation in a field unit. Thereafter, by subtracting this shot component from the raw focus map, the wafer table focus residual representing only the topographical variation in the pure wafer table was derived. Looking at the final focus residual map from which the offset was removed, while the center (0–130 mm) is flat, a distinct ERO phenomenon in which negative defocus rapidly deepens can be observed in the extreme outer (140–147 mm) region.

To isolate the mechanical ERO, focus errors unrelated to the table hardware are removed by this sequence.

(a) Focus normalized: The initial raw focus map and its radial scatter plot (0–147 mm) containing the focus fluctuations of the entire system.
(b) Focus shot component: modeled extraction and subtraction of systemic focus offsets and global tilt caused by reticle and scanner field leveling.
(c) Focus residual: The corrected focus map and radial scatter profile showing the topographic changes in the wafer table. This residual presents a flat center and a negative defocus at the extreme periphery (140–147 mm).

### 2.5. Mathematical Modeling of Dual-Edge Defocus Indicators

Based on the focus residual map extracted in Section 2.4, this study designed a mathematical framework of two indicators representing the statistical distribution and geometric shape to precisely monitor the ERO phenomenon occurring at the extreme outer edge of the wafer table (Table 2). As a data preprocessing step, approximately 10% of the focus data located in the 140–147 mm radius section were extracted as the region of interest from the thousands of data points collected across the entire wafer. Thereafter, by removing the focus offset value of the data and zeroing the center value of the residual to 0 nm, the deviation caused by the process was excluded and only the variation in the pure edge region was clearly separated.

The first indicator is a statistical indicator representing the macroscopic degradation of the 140–147 mm edge band. The extreme outer edge of the wafer is a section where severe measurement noise and outliers occur due to the overlap of equipment vibration, temperature fluctuation, and the global bow phenomenon of the wafer itself. Therefore, it is difficult to accurately track the table wear trend simply with an average or maximum/minimum value. Accordingly, this study introduced a Range Percentile model that excludes the influence of outliers by cutting off a certain ratio of the upper and lower parts. Considering the focus measurement performance level of this GOFM metrology system, it is essential to set an optimal cut-off ratio that can effectively filter out noise while fully reflecting the severity of actual physical wear. The statistical optimization process to derive this parameter is dealt with in detail through the long-term, time-series split evaluation in Section 3.2.

The second indicator is a geometric indicator (Slope × 3) quantifying the localized wear drop in the extreme outer 3 mm section. The physical friction stress generated by vacuum and clamping during wafer loading and unloading is most concentrated in the 144–147 mm section of the radius. To mathematically formulate the rapid shape deformation of this localized region, first, a 5th-order polynomial fitting was applied to the entire radius (0–147 mm) of the wafer. The 5th-order was selected through a quantitative sensitivity analysis against adjacent polynomial orders (3rd through 7th), summarized in Table 3, with all metrics normalized to the ground-truth edge-drop magnitude and the flat region evaluated over the full 0–140 mm range. The 3rd-order under-fits both flat and edge regions, recovering only 73.0% of the edge drop and yielding the largest edge-region RMSE (0.150). Even-order polynomials (4th, 6th) introduce spurious curvature as an artefact of their inherent radial symmetry, with the 4th-order showing the largest flat-region deviation (0.289) and the 6th-order exhibiting a localized peak near the edge-inflection zone (≈138 mm; deviation = 0.013). The 7th-order over-fits localized noise, more than doubling the flat-region deviation (0.019) compared to the 5th-order baseline while showing no improvement in edge-region RMSE (0.006). The 5th-order thus achieves the optimal balance across both regions, recovering 99.4% of the true edge drop with the minimum flat-region deviation (0.009) and edge-region RMSE (0.006). This empirical approach extends the polynomial-based ERO profile fitting methodology established in wafer-clamp designs [42].

**Figure 5:**
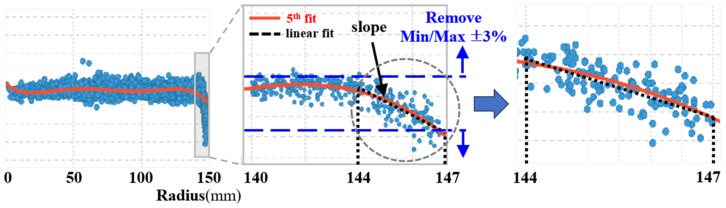
Mathematical modeling and extraction procedure of the geometric edge wear indicator.

This is a mathematical approach to simultaneously accommodate the flatness of the wafer center and the curvature change (inflection point) near the edge. Thereafter, only the 144–147 mm band drawing the steepest descending curve was extracted to derive a quasi-linear slope while excluding min/max ±3% outliers (Figure 5). The Second indicator, calculated by multiplying the instantaneous rate of change in the derived regression line by the evaluation bandwidth of 3 mm, represents an estimate of the total defocus drop generated by table wear in the corresponding 3 mm section beyond the meaning of a simple angle. Ultimately, this value is utilized as an important engineering reference point directly linked to the CD margin of semiconductor products in the future.

### 2.6. Data Collection over Multiple Tools and Time

To verify the effectiveness of the proposed GOFM-based focus metrology and dual-edge wearing indicators framework (Range Percentile and Slope × 3), long-term data were collected under the same protocol targeting multiple exposure equipment and wafer tables operating in an actual mass production line.

For each equipment and table combination, GOFM measurement was performed by periodically inputting an NPW, and the raw data of the focus residual map, 5th-order fitting curve, and dual-wear indicators were extracted through the previously defined mathematical algorithm. At the same time, actual product quality metrics such as the CD distribution of the edge region, edge yield, and pattern bridge defect occurrence rate were collected from the mass production wafer processed with the same process layer in the corresponding equipment, constructing a database that can cross-verify the correlation with the metrology indicators.

Data collection for this study was conducted over a long-term duration, and it was designed to include not only the simple wear progression section but also all the data of the section before and after maintenance, in which an aged wafer table is replaced with a new part. Through this multi-year, multi-tool-centered experimental design, this study prepared a foundation to structurally track the following three things:

(i) The long-term drift trajectory of the wear indicator according to the accumulated number of processed wafers and table usage time.
(ii) The physical edge defocus resilience before and after table replacement and whether the initial reference point (POR) of the product CD and yield are recovered.
(iii) The statistical distribution and deviation of mechanical wear patterns appearing across multiple equipment and tables.

The large-scale time-series metrology and product quality data collected in this way are used to perform the subsequent statistical optimization verification and confidence interval analysis, and are ultimately utilized as source data for Preventive Maintenance (PM) modeling that analyzes the critical defect arrival point.

## 3. Results

### 3.1. Benchmarking GOFM Against Conventional Focus Metrology

To detect the minute wear in the extreme edge region of the wafer table, the reliability of the metrology system itself must be established. In this study, based on the key performance indicators (KPIs) defined in Section 2.3, the existing industry-standard eDoF-based method and the proposed GOFM method were quantitatively compared and analyzed (Table 4).

As an analysis result, GOFM expanded the focus capture range by 25% compared to the baseline target (achieving a normalized index of 1.25) through ODMM pitch optimization. This enhancement ensures distortion-free measurement even in the extreme outer ERO topography, a region characterized by severe negative defocus. Furthermore, even under various dose variation environments, it suppressed measurement uncertainties, reducing the set/get residual by 22% compared to the eDoF method (normalized index of 0.70). It also controlled the ZAF to a normalized index of 0.66, well below the acceptance baseline of 1.00, thereby proving the structural robustness of the metrology tool.

An important result is the increase in the sensitivity and linearity indicator ($R^{2}$). The standard Ry tilt focus protocol reached an $R^{2}$ of 0.9851, whereas the GOFM method recorded 0.9993. This metrology performance enables the extraction and monitoring of local defocus in the 140–147 mm boundary. Conventional optics previously lacked the spatial resolution to evaluate this geometric domain.

### 3.2. Statistical Optimization and Correlation of Dual-Edge Indicators

To apply the dual-indicator (statistical distribution indicator and geometric ERO indicator) model designed earlier in Section 2.5 to mass production data, correlation analysis between indicators and parameter optimization were performed based on the focus residual data of the 140–147 mm section.

Especially for Range Percentile, which is a statistical indicator, the process of determining the upper and lower cut-off ratio is essential. For this, this study divided the percentile range into five cases from 99.7% to 95.0% and conducted a long-term time-series split evaluation. This split evaluation and the calculation of the correlation coefficient (R^2^) with the geometric indicator were performed based on long-term trend data accumulated over an extended period from multiple equipment groups input into actual HVM, in order to exclude the short-term bias of specific equipment and maximize statistical significance. The core criterion of the evaluation was placed on how effectively it blocks the measurement noise generated by this GOFM metrology system, while simultaneously and accurately following the long-term trend of actual geometric ERO (Slope × 3). Table 5 shows the quantitative optimization result of the corresponding split evaluation.

As shown in Table 5, under the 99.7% and 98.0% conditions where the cut-off range was set narrowly, noise was included in the indicator, leading to severe long-term trend distortion; consequently, the coefficient of determination (R^2^) with the geometric indicator (Slope × 3) was also derived low at 0.8 or less. Conversely, under the 96.0% and 95.0% conditions where the range was cut off too widely, noise was perfectly removed, but even the actual physical degradation signal was lost together, showing an over-filtered tendency where the correlation drops again.

**Figure 6:**
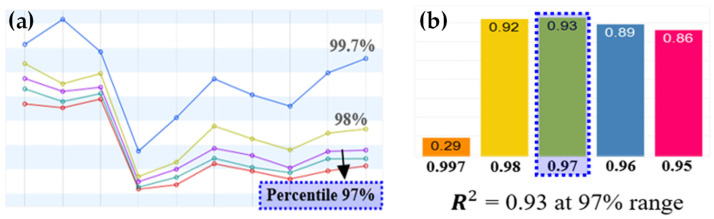
Statistical optimization and correlation analysis of the dual-edge-wear monitoring framework. (**a**) Focus residual stability in the extreme-edge region under varying outlier-rejection percentiles (95% to 99.7%). (**b**) Linear correlation between the geometric metric and the statistical boundary of Range Percentile.

As a result, the optimal balance of noise suppression and degradation signal preservation was achieved under the 97.0% condition where 3% of the data at both extremes was cut off. Under this condition, it was mathematically confirmed that the correlation coefficient between the statistical indicator and the geometric indicator (Slope × 3) derived from the extreme outer 3 mm section (144–147 mm) was maximized to $R^{2}=0.93$ (refer to Figure 6). According to this optimization result, which simultaneously reflects the limit of this measurement noise and the physical distortion of the actual wafer shape, this study confirmed Range Percentile 97% as the final statistical indicator.

### 3.3. Long-Term Validation in High-Volume Manufacturing (HVM)

**Figure 7:**
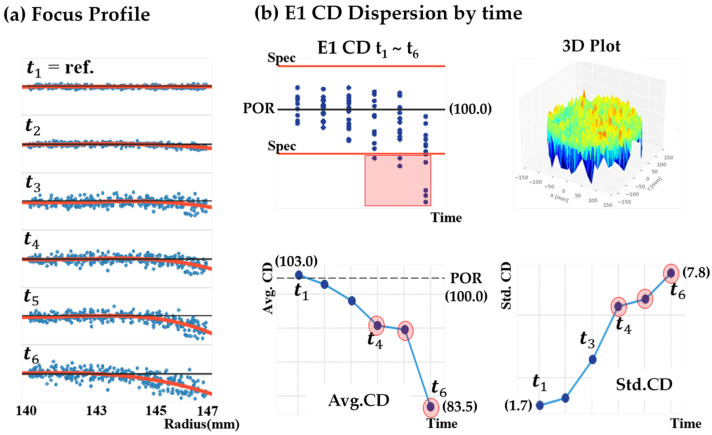
Chronological deterioration of the wafer table edge and its impact on process quality. (**a**) Downward trend of the extreme-edge focus profile (140–147 mm) across equipment operational history (t1 to t6). (**b**) Edge CD dispersion correlating with the focus decay.

To verify the impact that the dual-wear indicators have on actual product quality, long-term time-series data collected from multiple mass production equipment were analyzed. The chronological scope of the validation spans the complete maintenance lifecycle of the wafer table from early operation through end-of-life. The t1–t6 stages in Figure 7 represent normalized lifecycle phases across the multi-year HVM dataset. Absolute calendar dates and tool-specific operation hours are maintained within proprietary HVM specifications.

Figure 7 shows the long-term drift trajectory of the geometric wear indicator (Slope × 3) according to the accumulated table usage time. As the aging of the table progressed, the Slope × 3 indicator continuously increased. Completely synchronized with this progression, a phenomenon where the CD distribution inversion of the wafer edge region of actual products and the pattern bridge defect rate exceeded the limit spec was observed. To complement the qualitative trends, the statistical dispersion of the indicators was characterized on the POR-normalized scale (POR = 100) and is reported directly in Figure 7b. The mean edge CD declines from 103.0 at the early-life reference (t1) to 83.5 at the end-of-life (t6), while the corresponding inter-tool standard deviation broadens monotonically from 1.7 to 7.8 across the lifecycle, reflecting the divergence of wear states. The Range Percentile 97% indicator exhibits a correspondingly tighter dispersion throughout the lifecycle, consistent with its statistical noise-rejection design. Absolute CD and σ values remain within proprietary HVM specifications.

**Figure 8:**
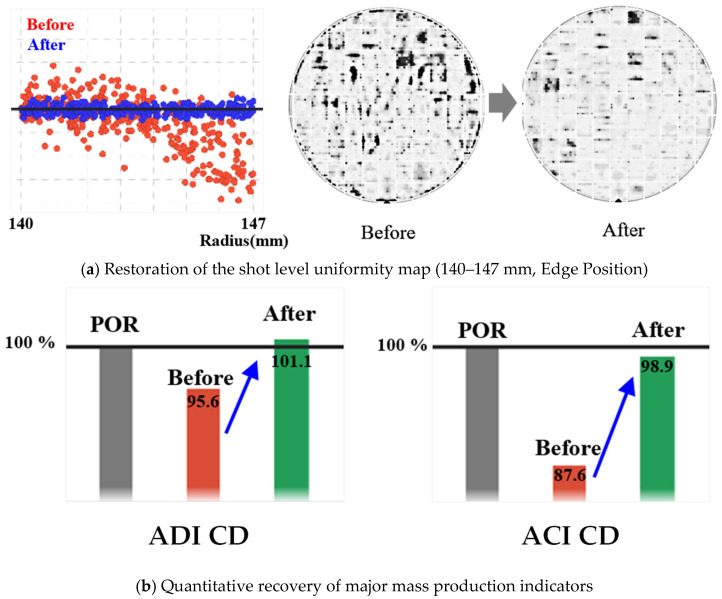
Process stability recovery and restoration after maintenance measures according to the condition-based maintenance trigger.

The validity of these indicators is more clearly proven through the comparison before and after equipment maintenance events. After replacing the wafer table that reached the threshold (after months of HVM mass production progression) due to physical wear, the focus of the wafer center and edge regions perfectly aligned, as can be confirmed in Figure 8. Accordingly, both the Slope × 3 indicator and the edge CD distribution distinctly recovered to a good POR level. Here, the initial POR state, which is the comparison reference point of this study, means the point when mechanical stabilization is completed through HVM operation in multiple identical equipment groups for part introduction and in-fab environment optimization, rather than simply right after mounting a new part. These findings confirm that the proposed edge wear monitoring system can directly contribute to the yield improvement of actual mass production lines based on high reliability and reproducibility. As quantified in Figure 8b, the ADI CD indicator recovered from a pre-maintenance value of 95.6 to 101.1 following the maintenance intervention, and the ACI CD indicator recovered from 87.6 to 98.9, on the POR-normalized scale (POR = 100). These values demonstrate a substantial restoration of edge CD uniformity attributable to the indicator-driven maintenance protocol. The underlying absolute production figures remain within proprietary HVM specifications.

### 3.4. Empirical Framework for Condition-Based Maintenance

**Figure 9:**
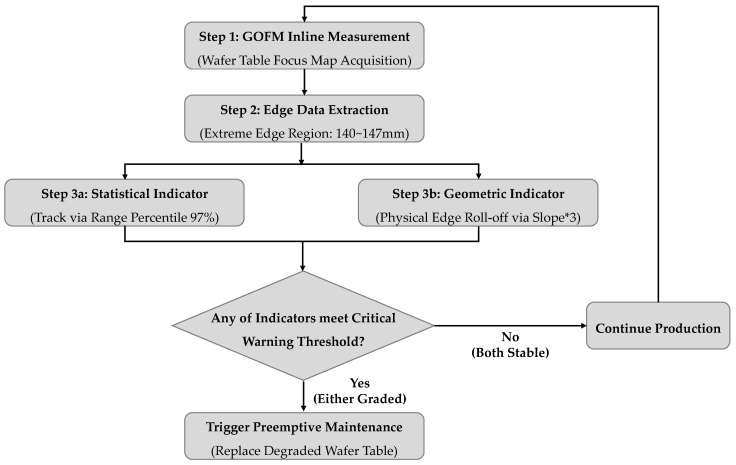
Workflow of the proposed model for the condition-based maintenance system integrated into the HVM environment.

Based on the long-term HVM data analysis results, this study derived a condition-based maintenance framework immediately applicable to HVM lines without a complex mathematical estimation model. This framework begins with in-line GOFM metrology and represents a series of sequential processes that extract the dual-indicator in parallel. The automated decision gate to which OR logic is applied triggers the preemptive replacement of the wafer table before CD defects occur, and when the equipment state is stable, it returns to the feedback loop to maintain continuous HVM production (Figure 9).

As verified in Section 3.3 earlier, the gradual increase in the Slope × 3 indicator according to the wafer table aging shows a very deterministic synchronization with the occurrence of bridge defects and yield degradation in the actual edge region. The 3 mm evaluation bandwidth (144–147 mm) was specifically chosen because this band, rather than only the outermost 1 mm slope, corresponds to the edge region that materially affects chip-level yield under the prevailing edge process rules, and the consistent monotonic co-evolution of the Slope × 3 trajectory with the edge CD anomaly probability across multiple HVM scanners and maintenance lifecycles establishes the indicator as a statistically reliable leading marker rather than a coincidental correlate.

This strong physical correlation means that the proposed geometric indicator (Slope × 3) can be utilized as a Statistical Process Control (SPC) variable representing the health indicator of the equipment. In this study, a specific Slope × 3 figure right before the edge CD distribution exceeds the process specification limit was set as the critical warning threshold. This threshold was determined through an empirical SPC-style back-mapping procedure: paired observations of (Slope × 3, edge CD distribution) collected across multiple tools and lifecycles were used to identify the Slope × 3 value at which the CD distribution begins to encroach upon a conservatively defined guard band below the CD upper specification limit, and the identical procedure was independently applied to the Range Percentile 97% indicator to derive its own threshold.

By integrating this threshold into the automated process control system inside the fab, equipment engineers can monitor the progression state of edge wear in real-time in a wafer unit. At the point when the Slope × 3 value reaches the corresponding threshold, the system automatically generates an alarm and triggers pre-emptive replacement.

This framework resolves the inefficiencies of conventional time-based preventive maintenance. Previous methods reacted after yield drops or replaced wafer tables based on fixed schedules despite their remaining useful life. Using empirical defocus trends as a maintenance trigger establishes a data-driven strategy. This approach maximizes equipment availability and resource utilization.

## 4. Discussion and Future Works

### 4.1. Advancing Edge-Resolved Focus Metrology with GOFM

This study proved the applicability of GOFM to quantify the ERO-based edge defocus induced by wafer table edge degradation in an HVM environment. Existing center/field-oriented focus evaluations, such as DBF and Focus-Exposure Matrix (FEM), are valid from a process window perspective, but there exists a structural limit in terms of spatial resolution to stably track the table-induced focus residual locally appearing in the 140–147 mm extreme outer region.

The GOFM-ODMM measurement architecture reconstructs the focus residual map. Isolating the table-induced residual at the extreme-edge coordinates separates mechanical hardware decay from routine process fluctuations. Performance metrics from these trials demonstrate the operational stability of the metrology framework in mass-production environments. This continuous monitoring expands upon isolated diagnostic checks. The inline optical method extends beyond patterning quality oversight by converting raw focus data into equipment health diagnostics and PM algorithms.

### 4.2. Linking Wafer Table Edge Degradation to Wafer Edge Defectivity

In the DUV immersion process, edge defocus leads not only to CD distribution degradation but also to fatal defects such as pattern bridges. This study investigated, through the analysis of long-term time-series data and multiple pieces of equipment, that one of the root causes of this defect occurrence mechanism lies in the mechanical wear of the equipment wafer table.

The long-term drift of the Slope × 3 metric correlates with the occurrence of CD defects and bridge formations. This relationship establishes the geometric parameter as a leading indicator. Single-variable monitoring carries risks of false positives and missed detections. To reduce these errors, the system combines the statistical Range Percentile 97% and the geometric Slope × 3 metric. An OR-logic protocol controls this dual-threshold framework and triggers an intervention when either parameter exceeds its limit. This methodology replaces reactive troubleshooting with condition-based maintenance. The system schedules preemptive equipment replacements at the warning threshold to maintain high-volume manufacturing throughput.

### 4.3. Robustness to Disturbances and Practical Deployment in HVM

High-volume manufacturing data contains environmental noise. Sources include global wafer bow, thermal drift, and stage dynamics. An edge monitoring metric needs sensitivity and operational robustness to process these signal variations. Our research applies a dual-filtering method. A Range Percentile 97% cutoff operates at the 140–147 mm edge boundary to remove transient outliers. A quasi-linear fitting evaluates the extreme-outer 3 mm section (144–147 mm) and extracts the Slope × 3 parameter. The correlation between these metrics reaches $R^{2}=0.93$. Tracking this data over time demonstrates that the model separates physical hardware degradation from temporary measurement noise.

This inline data flow (GOFM metrology → dual-edge wear indicator calculation → SPC monitoring) is easy to automate, and compared to the existing regular maintenance (time-based PM) method based on remaining useful life, it can minimize unnecessary equipment stoppage and part replacement.

From a computational-cost perspective, the framework operates on a periodic monitoring cadence: GOFM measurement is performed on a dedicated NPW approximately twice per month at each scanner equipment. Raw data and indicators are aggregated in an internal cloud-based server infrastructure deployed on multi-core x86 server-class processors with enterprise-grade memory and time-series storage. No GPU acceleration or dedicated inference hardware is required. The pipeline executes three closed-form deterministic operations per invocation: edge-region filtering, fifth-order polynomial fitting with Slope × 3 extraction, and Range Percentile 97% reduction. Each invocation completes on the order of seconds. This profile is substantially lower than ML-based alternatives requiring dedicated inference hardware and periodic model retraining, while providing edge-localized resolution beyond the eDoF baseline. Detailed configurations remain within proprietary HVM specifications.

Several limitations of the framework should be acknowledged. First, validation is restricted to 300 mm ArF DUV immersion scanners, and transfer to EUV systems with reflective optics and electrostatic chucks requires further structural reinterpretation, as elaborated in Section 4.4 [43,44]. Second, the dual-indicator architecture targets mechanical edge wear specifically, and other failure modes such as chuck contamination, stage-vibration anomalies, or lens-aberration drift would require complementary monitoring variables. Third, the Range Percentile 97% cut-off and the OR-logic warning thresholds were derived empirically via SPC-style back-mapping against in-house CD specifications, and may require recalibration for different process layers or device technologies. Fourth, the current framework treats the Slope × 3 trajectory as a deterministic leading indicator without probabilistic RUL estimation, which is addressed as the PdM extension in Section 4.4.

### 4.4. Future Works

While this study focused on DUV-based immersion lithography systems, we plan to extend this research in two key directions.

First, the proposed framework will be generalized and adapted to alternative lithography platforms. The developed GOFM-based residual analysis algorithm and dual-indicator architecture can, in principle, be transferred to monitor chuck contamination and degradation in Extreme Ultraviolet (EUV)-based lithography tools. However, since EUV systems rely on mirror-based reflective configurations rather than transmissive optics, a fundamental redesign considering the reticle and mask layout is required, which will be comprehensively addressed in a subsequent standalone investigation [4,45].

Second, the current framework will be expanded from CBM to a proactive predictive maintenance (PdM) paradigm [46]. We are currently developing a methodology to probabilistically forecast the RUL by utilizing the time-series metrology profiles obtained via GOFM. To achieve this, the potential for advanced predictive monitoring will be verified by correlating the metrology results with high-frequency equipment parameters, such as the wafer loading position and the time-series vertical motion profiles of the three lift pins supporting the wafer on the table.

## 5. Conclusions

This study investigated how long-term mechanical wear at the wafer table edge affects patterning yield in the DUV immersion lithography process. Based on this analysis, we constructed a condition-based maintenance framework to support preemptive maintenance action. The main conclusions are as follows.

First, GOFM was applied to the extreme edge region of 140–147 mm. This approach was used to overcome the limitation of conventional center-oriented optical metrology. As a result, the wafer table focus residual was successfully separated and quantified with high precision even under process noise and variation.

Second, a dual-indicator model was proposed for diagnosis of edge degradation. This model combines a statistical distribution indicator, Range Percentile 97%, with a geometric roll-off indicator, Slope × 3. HVM time-series data collected from multiple tools were analyzed. The results confirmed high correlation and long-term drift traceability between the two indicators. The indicators also showed deterministic warning capability before CD defects and pattern bridge defects occurred on production wafers.

Third, an automated decision gate based on OR logic was designed. This gate takes action when either of the two indicators reaches the threshold. Through this framework, the risks of over-maintenance and sudden failure in conventional preventive maintenance can be reduced. At the same time, the system enables wafer table replacement before CD defects occur on actual wafers. This supports improved equipment uptime and extreme edge yield.

In conclusion, this study presented a methodology that extends metrology toward hardware health monitoring and condition-based maintenance in semiconductor manufacturing. This established framework not only serves as a baseline for monitoring chuck degradation in EUV and next-generation patterning equipment, but also paves the way for advanced predictive maintenance (PdM) models by integrating multivariate sensor data with wafer loading configurations and lift-pin motion profiles.

## Figures and Tables

**Table 1:** Summary of lithography tools, metrology, and environmental conditions.

**System & Tooling**	**Environmental Control (Lot Start to End)**
Scanner: ASML Advanced DUV Immersion Scanner (ASML, Veldhoven, The Netherlands)	Temperature: Standard Cleanroom Conditions
Metrology: ASML Inline Scatterometry Tool (ASML, Veldhoven, The Netherlands)	Pressure: Standard Cleanroom Conditions
**Optical & Measurement Setup**	**Material & Process (POR)**
Illumination: quad pole type	Wafer: 300 mm Bare Si (NPW)
NA: 1.35 (Default)	Reticle: ODMM-dedicated
Measurement Points: thousands pts @ Full Wafer	Photoresist/Dose: Proprietary HVM

Note: detailed mark geometry, exact dose settings, and photoresist chemistry are maintained within proprietary POR boundaries for HVM.

**Table 2:** Mathematical definitions and parameters for the dual-edge defocus indicators.

Parameter	Mathematical Definition	Value/Condition	Rationale
Edge region of Interest	$R_{edge}=\{r_{i}|140\leq r_{i}\leq 147\;\mathrm{mm}\}$	N≈10%	Filtering edge-specific data from full wafer
Data preprocessing	$F_{norm}(r_{i})=F(r_{i})-\mathrm{Median}(F_{edge})$	Center = 0 nm	Removal of focus offset and centering of residuals
Statistical indicator: Range Percentile X	$I_{stat}=P_{X}(F_{norm})-P_{100-X}(F_{norm})$	parameter *X* to be selected	Prevention of data distortion due to Meas. noise, outliers
Geometric indicator: Slope×3	$P(r)=\sum_{k=0}^{5}a_{k}r^{k}$ $I_{geom}=\left(\frac{dP(r)}{dr}\right)\times 3\;\mathrm{mm}$	region: 144≤r≤147 mm	Quantifying the steepest ERO gradient within the 3 mm

**Table 3:** Polynomial-order sensitivity analysis for baseline ERO profile fitting, normalized to the ground-truth edge-drop magnitude.

*n*-Order Fit	Recovered Edge Drop	Flat-Region Max Deviation (0–140 mm)	Edge-Region RMSE (140–147 mm)	Decision
3rd-order	0.730	0.252	0.150	Under-fit
4th	0.871	0.289	0.072	Even-order
5th	0.994	0.009	0.006	Optimal
6th	1.002	0.013	0.008	Even-order
7th	0.991	0.019	0.006	Over-fit

**Table 4:** Benchmarking GOFM against conventional focus metrology KPI (normalized to HVM baseline targets).

KPI (from Section 2.3)	eDoF	GOFM	Improvement (Δ)	Acceptance/Target (Baseline)
Focus capture range	1.00	1.25	+25% wider	≥1.00
Set/get residual	0.92	0.70	−22% lower	≤1.00
ZAF (Zero Asymmetry Focus)	N/A	0.66	N/A	≤1.00
Sensitivity/linearity (R^2^)	0.9851	0.9993	+0.0142	1.0000

**Table 5:** Statistical optimization of the Range Percentile indicator based on empirical split evaluation (Evaluated sample: year-based long-term data across HVM scanners).

Range Percentile	Cut-Off Ratio	Sensitivity to Noise	Trend Distortion in Time-Series	Correlation with Slope × 3 (R^2^)	Optimization Decision
99.7%	0.30%	High	Severe distortion	0.76	Noise-dominated
98.0%	2.00%	Moderate	Moderate distortion	0.84	Unstable trend
97.0%	3.00%	Low	Stable (less distortion)	0.93	Optimal balance
96.0%	4.00%	Very Low	Signal loss (damped trend)	0.90	Over-filtered
95.0%	5.00%	Very Low	Signal loss (damped trend)	0.86	Over-filtered

## Data Availability

The data presented in this study are available on request from the corresponding author due to proprietary restrictions.

## Abbreviations

ArF	Argon Fluoride
ACI	After Cleaning Inspection
ADI	After Development Inspection
CD	Critical Dimension
CDU	Critical Dimension Uniformity
DBF	Diffraction-Based Focus
DoF	Depth of Focus
DUV	Deep Ultraviolet
eDoF	Exact Depth of Focus
ERO	Edge-Roll-Off
EUV	Extreme Ultraviolet
FDC	Fault Detection and Classification
FEM	Focus-Exposure Matrix
GOFM	Geometry-Based Optical Focus Metrology
HVM	High-Volume Manufacturing
KPI	Key Performance Indicator
MES	Manufacturing Execution System
ML	Machine Learning
NA	Numerical Aperture
NPW	Non-Production Wafer
ODMM	Optical Diffraction Metrology Mark
OPC	Optical Proximity Correction
PM	Preventive Maintenance
POR	Process of Record
PR	Photoresist
RET	Resolution Enhancement Techniques
RUL	Remaining Useful Lifetime
SEM	Scanning Electron Microscope
SPC	Statistical Process Control
SWA	Side Wall Angle
ZAF	Zero Asymmetry Focus
